# Supplementary material for: Self-protecting CoFeAl-layered double hydroxides enable stable and efficient brine oxidation at 2 A cm−2
Source: Nat Commun. 2024 Jun 3;15:4712. doi: 10.1038/s41467-024-49195-z (PMC11148009; doi:10.1038/s41467-024-49195-z)
Supplement: Supplementary file 1 — Supplementary Information [file 41467_2024_49195_MOESM1_ESM.pdf]

Self-protecting CoFeAl-layered double hydroxides Enable Stable and Efficient Brine Oxidation at 2 A cm^{-2}

Wei Liu[1], Jiage Yu[1], Tianshui Li[1], Shihang Li[1], Boyu Ding[1], Xinlong Guo[1], Aiqing Cao[1], Qihao Sha[1], Daojin Zhou[1, *], Yun Kuang[2, *], Xiaoming Sun[1, *]

[1] State Key Laboratory of Chemical Resource Engineering, College of Chemistry, Beijing University of Chemical Technology, Beijing 100029, China. [2] Ocean Hydrogen Energy R&D Center, Research Institute of Tsinghua University in Shenzhen, Shenzhen 518057, China.

Corresponding authors

Xiaoming Sun, email: sunxm@mail.buct.edu.cn

Daojin Zhou, email: zhoudj@buct.edu.cn

Yun Kuang, email:kuangy@tsinghua-sz.org

Supplementary Information Notes

Supplementary Note 1.

In the hydrogen production process through electrolysis using pure water, it is common to add a certain amount of pH adjuster to reduce the electrolyte's resistance and accelerate the kinetics of the hydrogen evolution reaction (HER) and oxygen evolution reaction (OER). For instance, in traditional alkaline water electrolysis (ALK), 20-30 wt.% of an alkaline electrolyte is added to pure water. Similarly, alkaline seawater electrolysis, which is the direct electrolysis of seawater under alkaline conditions, follows a similar approach.

On the anode side of seawater electrolysis, the presence of chloride ions leads to a competition between the oxygen evolution reaction (OER) and the chlorine evolution reaction (CER). The reaction equations and theoretical potentials are as follows:

$$4OH^- \rightarrow 2H_2O + O_2 + 4e^- \quad E^o = 1.23\ V - 0.059pH \tag{1}$$

$$2Cl^- \rightarrow Cl_2 + 2e^- \quad E^o = 1.36\ V \tag{2}$$

$$Cl^- + 2OH^- \rightarrow ClO^- + H_2O + 2e^- \quad E^o = 1.72\ V - 0.059pH \tag{3}$$

In 2015, Peter Strasser and colleagues explored the optimal conditions for seawater electrolysis-based hydrogen production using the equations mentioned above and the Pourbaix diagram.[1] At a temperature of 25°C and a Cl^- concentration of 0.5 M in the electrolyte, the pH of the electrolyte needs to be greater than 7.5 to achieve the widest potential window of 480 mV between OER and CER. In other words, within this pH range, there is no possibility of the CER side reaction occurring as long as the anodic OER overpotential is lower than 480 mV.

Supplementary Note 2.

During the continuous seawater electrolysis process, the net consumption of reactants on both the cathode and anode sides is water, while the electrolyte replenishment comes from fresh seawater. As a result, the concentration of NaCl continuously increases until saturation. The typical saturation concentration of NaCl is around 5.5 M. When performing alkaline seawater electrolysis for hydrogen production with only 1 M KOH added, the concentration disparity between OH^- and Cl^- at this NaCl saturation level can lead to severe electrode corrosion. In 2020, Sun et al. developed a hydrogen-oxygen-salt co-production electrolysis process based on the co-ion effect to address the continuous alkaline seawater electrolysis issue.[2] By adding 6 M NaOH to seawater (approximately 20 wt.%, consistent with the alkali concentration used in traditional alkaline electrolysis, ALK), the saturation concentration of NaCl is dramatically reduced to around 2.8 M due to the common ion effect. Under this condition, the concentration difference between OH^- and Cl^- is approximately 1:0.5, effectively preventing corrosion and producing pure NaCl crystals during the electrolysis process.

Supplementary Information Figures

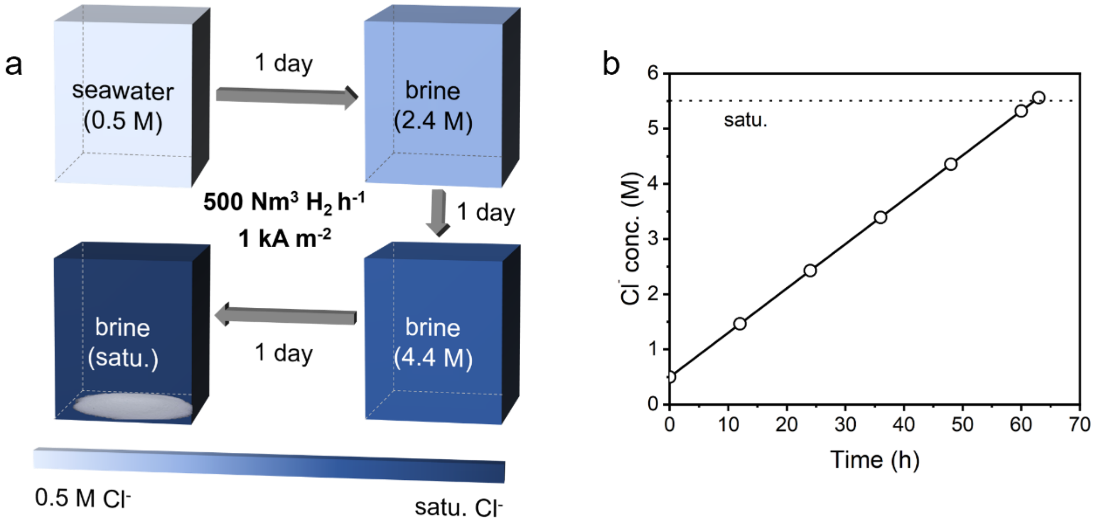

Supplementary Figure 1. (a) The schematic diagram of continuous seawater electrolysis and (b) the concentration (conc.) of Cl⁻.

Assuming a current density of 10 kA m^{-2}, a commercial alkaline electrolysis water device with a hydrogen production capacity of 500 Nm^3 h^{-1} and a liquid storage volume of 2500 L was used to calculate the cumulative concentration of NaCl. The water consumption rate can be easily obtained from the hydrogen production rate, which is 401.79 L h^{-1}. Feeding seawater to maintain the electrolyte volume constant will result in an increase in NaCl concentration of 0.08 M per hour. The calculation formula is as follows:

$$\Delta C = {C_0 \times \Delta V}/{V_0} = {0.5\ \mathrm{M} \times 401.79\ \mathrm{L\ h^{-1}}}/{2500\ \mathrm{L}} = 0.08\ \mathrm{M\ h^{-1}} \quad (4)$$

In which, ΔC represents the increase rate of NaCl concentration, C_0 and V_0 represent the initial NaCl concentration and electrolyte volume, and ΔV represents the volume of electrolyte consumed.

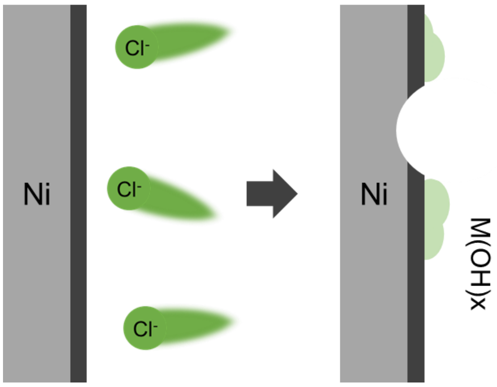

Supplementary Figure 2. The corrosion mechanism of commercial nickel foam electrode.

In the OER process, Cl^- are affected by the electric field and migrate to the anode surface, where they undergo corrosion after adsorption. This process mainly involves the following three steps[3]:

The first step—Adsorption of Cl^- by surface polarization:

$M + Cl^- \rightarrow MCl_{ads} + e^-$

The second step—Dissolution by further coordination

$MCl_{ads} + Cl^- \rightarrow MClx^-$

The third step—Conversion from chloride to hydroxide

$MClx^- + OH^- \rightarrow M(OH)x + Cl^-$

In the electrolyte containing brine, the competitive adsorption OH^- on the electrode surface is hindered, leading to increased susceptibility to corrosion.

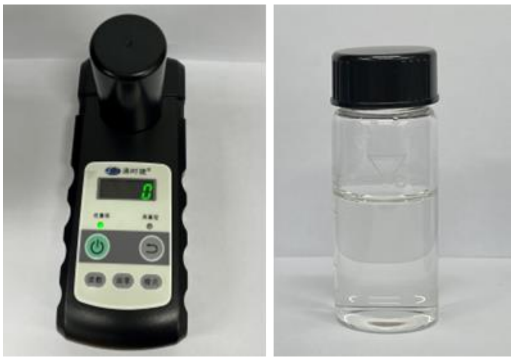

Supplementary Figure 3. Active chlorine test of 1 M NaOH + 3 M NaCl electrolyte after being electrooxidized by Ni foam anode.

The commercial active chlorine detector was used to detect the electrolyte after the chronopotentiometry (CP) response. The detector displays the concentration of active chlorine (0 ppm), and the color of the electrolyte remains unchanged after adding commercial color indicator and commercial pH adjuster. Indicated there is no generation of active chlorine species during the process.

Although the Ni foam anode experienced severe corrosion within 0.2 h, experimental results showed no significant chlorine oxidation reaction (COR).

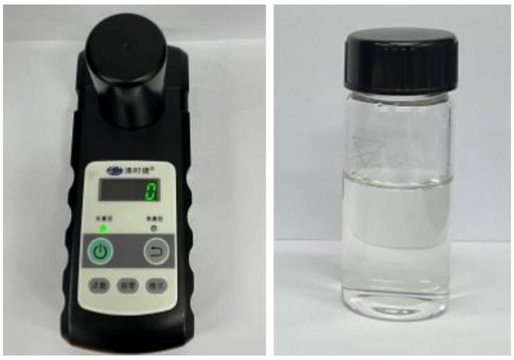

Supplementary Figure 4. Active chlorine test of 20 wt.% NaOH + saturated (satu.) NaCl electrolyte after being electrooxidized by Ni foam anode.

The detector displays the concentration of active chlorine (0 ppm), and the color of the electrolyte remains unchanged after adding commercial color indicator and commercial pH adjuster. Indicated there is no generation of active chlorine species during the process.

After increasing the concentration of NaOH, Ni foam showed better corrosion resistance. Although some green $Ni(OH)_2$ precipitates appeared on the surface after 1 h, there was no fracture. And experimental results showed no significant COR. This also indicates that Ni foam has excellent oxygen evolution selectivity and is one of the most promising substrates for anodes in concentrated brine electrolysis.

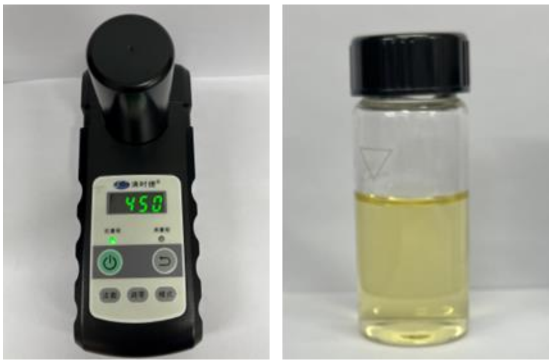

Supplementary Figure 5. Active chlorine test of 1 M NaOH + 3 M NaCl electrolyte after being electrooxidized by IrO_2 anode.

The detector displays the concentration of active chlorine (450 ppm), and the color of the electrolyte changed to yellow after adding commercial color indicator and commercial pH adjuster.

This indicates that IrO_2 has decent OER activity but also excellent COR performance. Coupled with its high price, it is not suitable as an anode material for concentrated brine electrolysis.

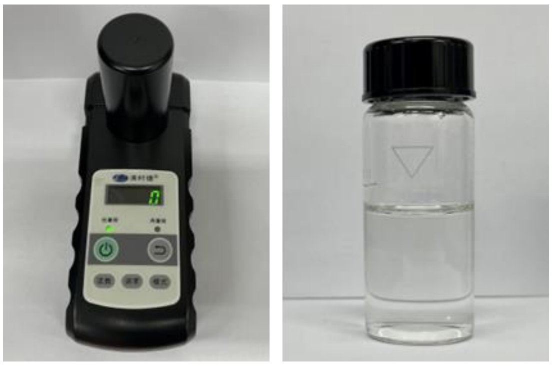

Supplementary Figure 6. Active chlorine test of 20 wt.% NaOH + saturated (satu.) NaCl electrolyte after being electrooxidized by IrO_2 anode.

The detector displays the concentration of active chlorine (0 ppm), and the color of the electrolyte remains unchanged after adding commercial color indicator and commercial pH adjuster. Indicated there is no generation of active chlorine species during the process.

After increasing the concentration of NaOH, IrO_2 showed excellent OER selectivity. This also indicates that the high concentration NaOH is the most promising additives for concentrated brine in electrolysis.

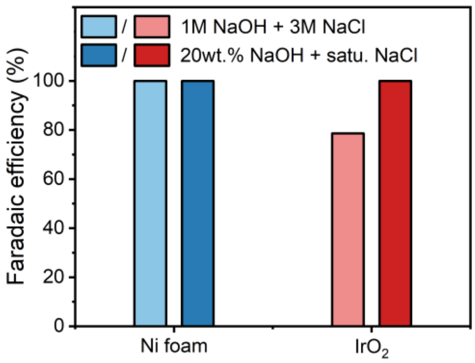

Supplementary Figure 7. The corresponding Faradaic efficiency of the commercial electrodes' chronopotentiometry response at 0.2 A cm^{-2}.

When commercial IrO_2 and commercial nickel foam anodes are used in electrolytes containing brine, these anodes are prone to undergo chlorine oxidation side reactions, leading to the formation of toxic substances.

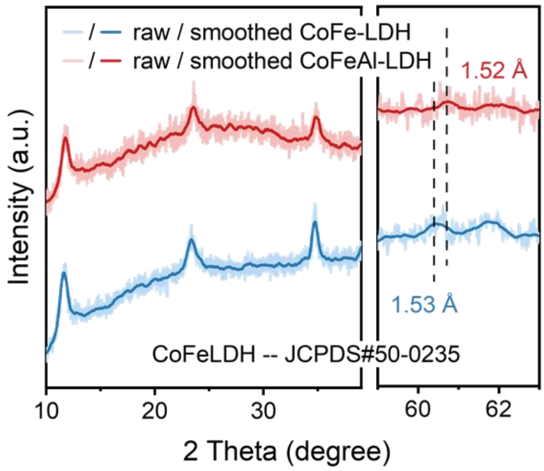

Supplementary Figure 8. XRD patterns of CoFe-LDH and CoFeAl-LDH.

The structure of CoFe-LDH and CoFeAl-LDH can be confirmed by X-ray diffraction (XRD), in which the diffraction peaks align well with the reported lattice planes of CoFe-LDH (JCPDS#50-0235). Additionally, it can be observed from the diffraction peaks at approximately 60.6° that CoFeAl-LDH exhibits a deviation towards higher angles compared to CoFe-LDH. The lattice spacing of CoFeAl-LDH (1.52 A) determined using Bragg's law was found to be slightly reduced in comparison to that of CoFe-LDH (1.53 A). These findings indicate that Al^{3+} with smaller radii than Fe^{3+} were incorporated into the CoFe-LDH structure. Note that the unit of intensity is arbitrary unit (a.u.).

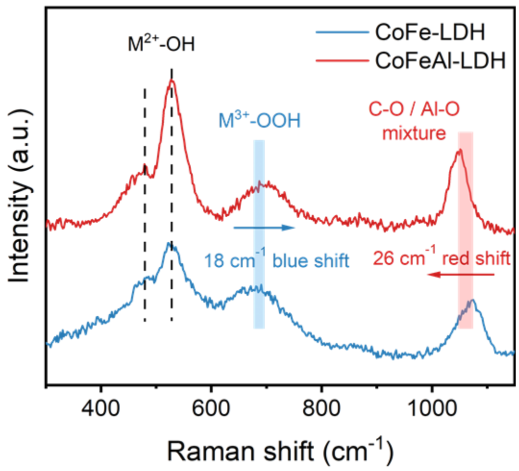

Supplementary Figure 9. Raman spectrum of CoFe-LDH and CoFeAl-LDH.

The Raman spectrum demonstrated a pair of peaks with fixed positions in the 400-600 cm^{-1} region before and after the introduction of Al^{3+} species into LDH. This indicated that the Co^{2+}-OH bond remains unchanged.[4] Compared to CoFe-LDH, the peak attributed to M^{3+}-O bending at around 690 cm^{-1} in CoFeAl has undergone a blue shift of 18 cm^{-1}, and the peak attributed to C-O at approximately 1050 cm^{-1} has experienced a red shift of 26 cm^{-1}. Note that the blue shift occurs because the Al-O bond is shorter than the Fe-O bond, leading to a higher bending vibrational frequency.[5,6] On the other hand, the redshift occurs due to the longer Al-O bond compared to the C-O bond, resulting in a lower stretching vibrational frequency.[7] These shifts further indicate the successful synthesis of CoFeAl-LDH.

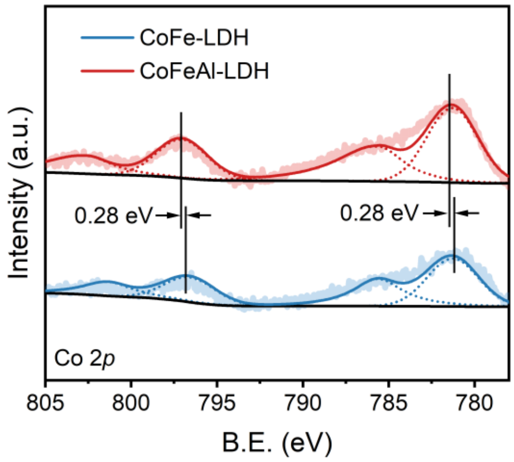

Supplementary Figure 10. Co 2*p* High-resolution XPS of CoFe-LDH and CoFeAl-LDH.

The High-resolution X-ray photoelectron spectrum (XPS) of Co 2*p* was performed to investigate the elemental oxidation states, which shows a pair of peaks located at approximately 781 eV and 797 eV, corresponding to Co^{2+} $2p_{3/2}$ and $2p_{1/2}$ spin orbitals, respectively.[8] And the other are associated to satellite peaks. It is evident that the introduction of Al^{3+} into CoFe-LDH causes a shift in the Co 2*p* signal towards higher binding energy, approximately 0.5 eV. This phenomenon suggests that Al (1.61) has a weaker electronegativity than Fe (1.83), leading to an increased of the electron cloud density near the Co atom (1.88) and thus maintaining a lower oxidation state. This outcome further confirms the successful synthesis of CoFeAl-LDH.

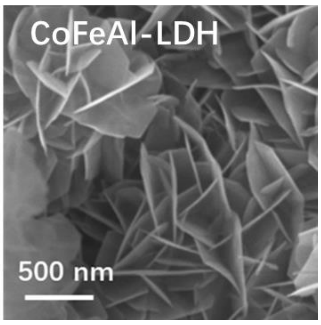

Supplementary Figure 11. SEM image of CoFeAl-LDH.

Scanning electron microscope (SEM) images show the similar 2D nanoarray morphologies of the as-prepared CoFeAl-LDH. These structures have long been known to exhibit hydrophilicity in electrolytic solutions, which facilitates the mass transport of O_2 products.[9,10]

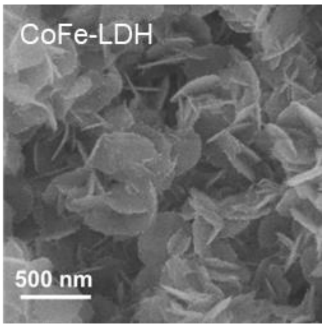

Supplementary Figure 12. SEM image of CoFe-LDH.

CoFe-LDH exhibits a nanosheet array morphology similar to that of CoFeAl-LDH.

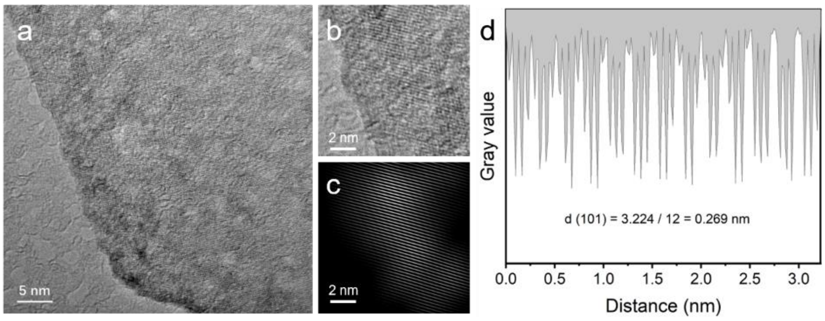

Supplementary Figure 13. (a) (b) High-resolution transmission electron microscope (HRTEM) images of CoFeAl-LDH and (c) (d) the identification process.

As shown in Figure a, the sample exhibits a characteristic hexagonal plate-like structure of LDH. After magnification and filter, Figure b and c were obtained, from which distinct lattice fringes can be observed. By measuring the width, it was found that they are consistent with the lattice spacing of the (101) crystal plane of CoFe-LDH in the database.

HRTEM image demonstrating a lattice spacing of 0.269 nm, assigned to (101) of CoFeAl-LDH.

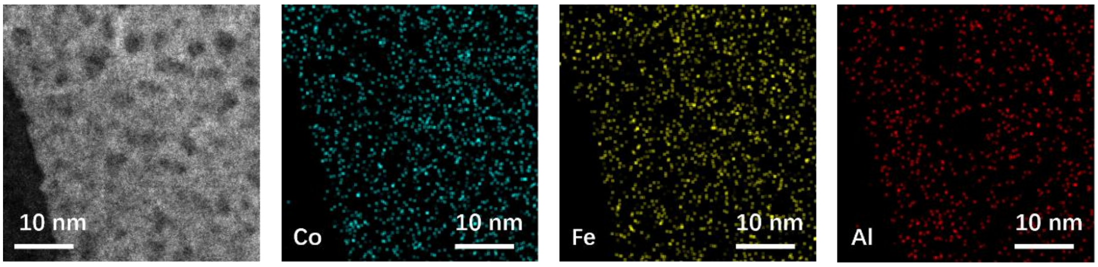

Supplementary Figure 14. HAADF-STEM image of CoFeAl-LDH and the corresponding EDS-elemental mapping for Co, Fe and Al.

High-angle annular dark field scanning transmission electron microscope (HAADF-STEM) images and the corresponding Energy Dispersive X-ray Spectroscopy (EDS) elemental mapping is utilized, showing that Co, Fe and Al elements are uniformly dispersed over a single nanosheet.

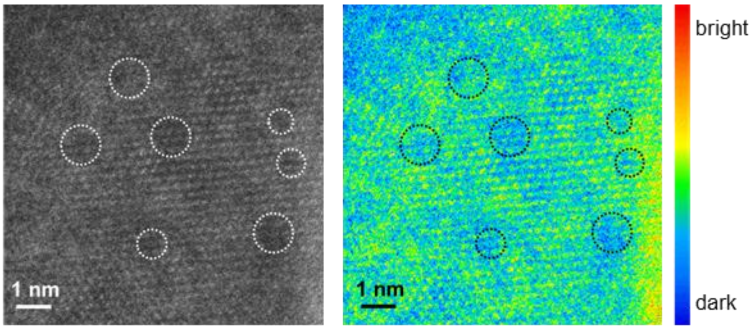

Supplementary Figure 15. High-angle annular dark field scanning transmission electron microscope (HAADF-STEM) image of CoFeAl-LDH and the identification process.

The darker areas of the atomic-scale HAADF-STEM images are assumed to represent Al-sites, as elements with lower atomic numbers typically exhibit lower contrast.[11] The image has been processed with pseudo-color to distinguish the Al^{3+}-sites. As shown in the images, the areas surrounded by dashed circles represent these sites. The atomic-scale image of HAADF-STEM provides further evidence of the homogeneous dispersion of Al atoms.

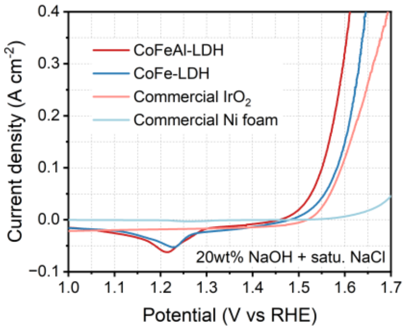

Supplementary Figure 16. Polarization curves of CoFeAl-LDH, CoFe-LDH, IrO_2 and Ni foam in 20 wt.% NaOH + satu. NaCl.

The CoFeAl-LDH electrode required only 256 mV overpotential to achieve 10 mA cm^{-2}. The performance is superior compared to CoFe-LDH (279 mV), IrO_2 (306 mV) and Ni foam (392 mV). It is worth noting that the array electrodes showed higher current growth rates. This is due to the hydrophobicity of the array structure, which can facilitate mass transfer of O_2 species.

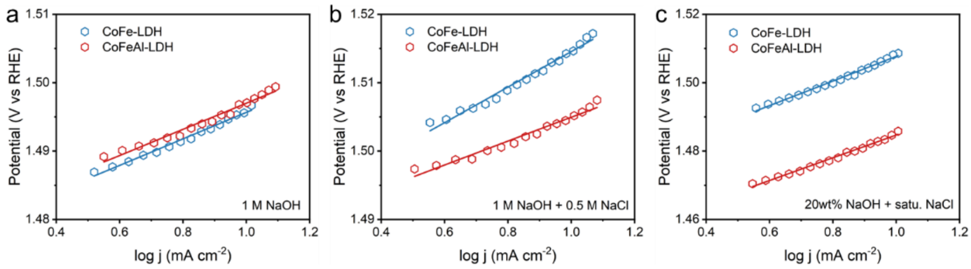

Supplementary Figure 17. Tafel plots of CoFeAl-LDH, CoFe-LDH, IrO_2 and Ni foam in (a) 1 M NaOH, (b) 1 M NaOH + 0.5 M NaCl and (c) 20 wt.% NaOH + satu. NaCl.

The Tafel slopes of CoFe-LDH in these electrolytes are 19.4, 26 and 36 mV dec^{-1}, respectively. It can be observed that as the Cl^- concentration increases, the OER kinetics of CoFe-LDH become increasingly sluggish. This result proves that Cl^- adsorbs on the active sites of the electrode, leading to poisoning of these sites and loss of OER activity.

However, as for CoFeAl-LDH, the Tafel slopes are 19.1, 17.5 and 23.2 mV dec^{-1}, respectively. Demonstrated that the OER kinetics cannot be affected by Cl^-.

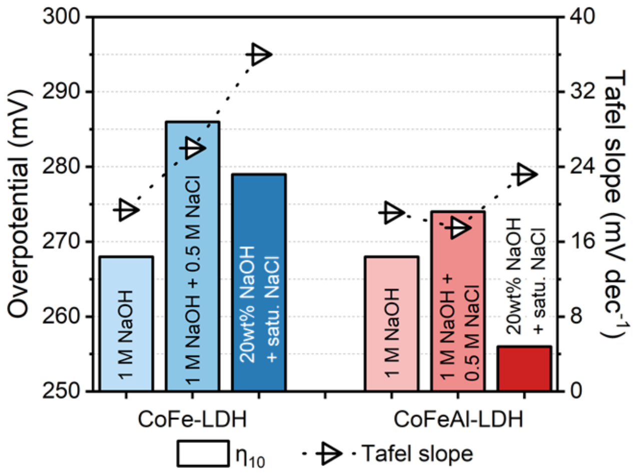

Supplementary Figure 18. Overpotential at 10 mA cm^{-2} and Tafel slope.

These two electrodes exhibited similar activity in low-concentration NaOH, but CoFeAl-LDH exhibited significantly better performance in high-concentration NaOH. According to previous reports, this improvement may be attributed to the dissolution of the amphoteric metal Al^{3+} in the CoFeAl-LDH electrode, which facilitates the exposure of more active sites.

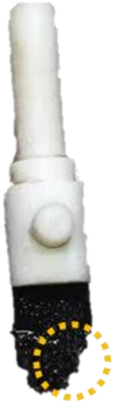

Supplementary Figure 19. Photograph of CoFe-LDH electrode after being corroded.
The CoFe-LDH electrode corroded and lost its ability to catalyze OER within 50 h in 20 wt.% NaOH + satu. NaCl electrolyte under 0.2 A cm^{-2}.

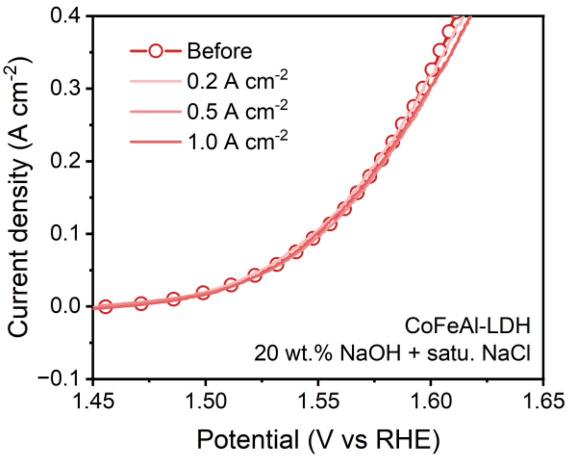

Supplementary Figure 20. Polarization curves of CoFeAl-LDH before and after the stability test at 0.2 A cm^{-2}, 0.5 A cm^{-2} and 1.0 A cm^{-2}, respectively.

These polarization curves were tested in 20% NaOH + satu. NaCl. The results showed that under 0.2-1.0 A cm^{-2}, after 1000 h of stability testing, there was no significant performance degradation of the electrodes. Note that the electrolyte used for stability testing was also 20% NaOH + satu. NaCl.

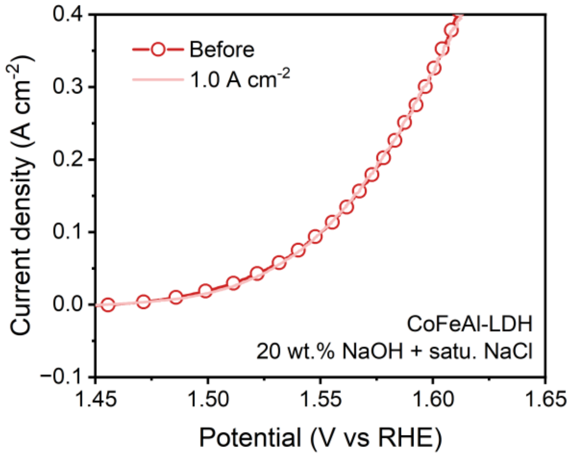

Supplementary Figure 21. Polarization curves of CoFeAl-LDH before and after 300 hours stability test at 1.0 A cm^{-2}.

These polarization curves were tested in 20% NaOH + satu. NaCl. The results showed that under 1.0 A cm^{-2}, after 500 h of stability testing, there was no significant performance degradation of the electrodes. Note that the electrolyte used for stability testing was 20 wt.% NaOH + 6-fold concentrated seawater.

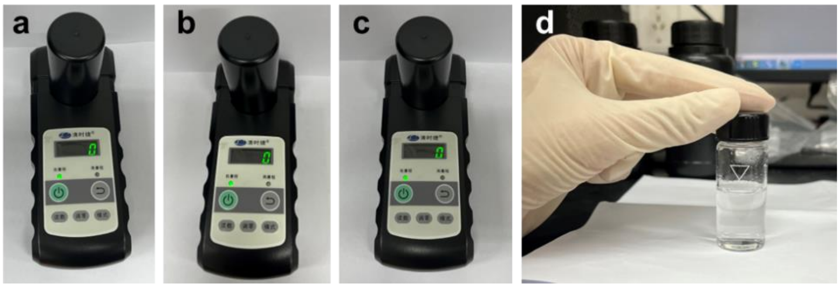

Supplementary Figure 22. Active chlorine tests of 20 wt.% NaOH + 6-fold concentrated seawater electrolyte after the stability test under (a) 1.0 A cm^{-2}, (b) 1.5 A cm^{-2} and (c) 2.0 A cm^{-2}. (d) The photograph of electrolyte with commercial color indicator and commercial pH adjuster.

The detector displays the concentrations of active chlorine (0 ppm), and the color of the electrolyte remains unchanged after adding commercial color indicator and commercial pH adjuster. Indicated there is no generation of active chlorine species during the stability test under 1.0, 1.5 and 2.0 A cm^{-2}.

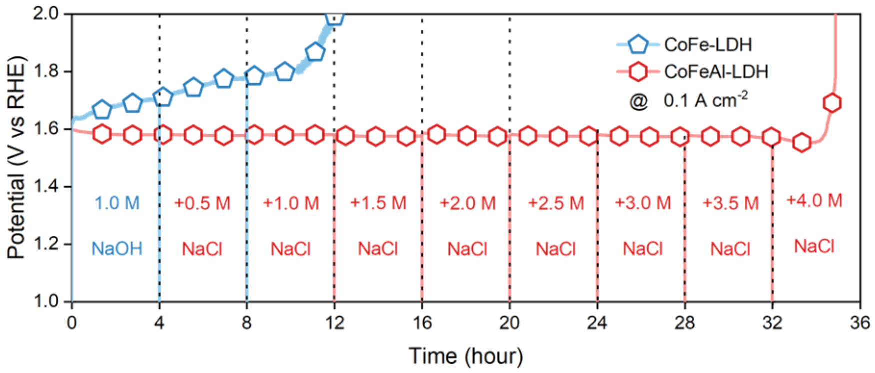

Supplementary Figure 23. The CP response of CoFe-LDH and CoFeAl-LDH at 0.1 A cm^{-2}.

In order to determine the maximum Cl^- concentration that the CoFeAl-LDH electrode can withstand, 1M NaOH electrolyte was utilized. Periodic additions of NaCl (0.5 M each 4 h) were introduced into it, and the current density of 0.1 A cm^{-2} was chosen for the experiment. It can be observed that the CoFe-LDH electrode began to decline when the Cl^- to OH^- ratio reached 0.5/1. But the CoFeAl-LDH electrode showed higher tolerance and could withstand a ratio of up to 4/1.

It is worth mentioning that these extreme conditions, where the Cl^- to OH^- ratio reaches such levels, are not typically encountered in industrial electrolysis processes, which commonly use 20 wt.% NaOH.

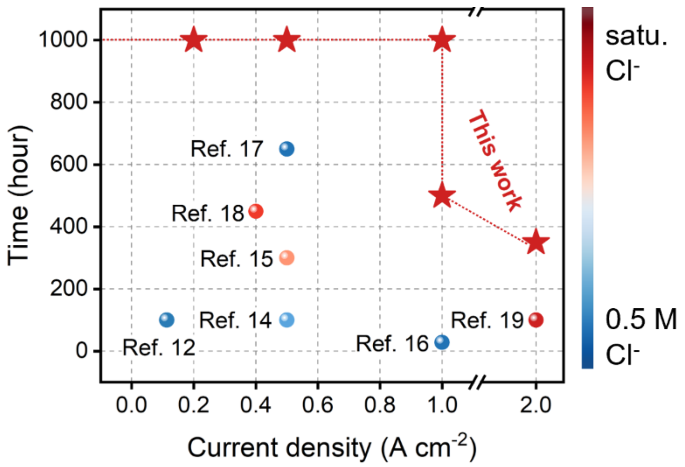

Supplementary Figure 24. The stability of CoFeAl-LDH compared with some recently reported electrodes.[12–19]

CoFeAl-LDH has demonstrated exceptional stability even at ultra-high current densities, such as 2 A cm^{-2}, in concentrated seawater. This remarkable performance sets CoFeAl-LDH apart from previous electrodes and highlights its potential for practical applications in the continuous seawater electrolysis.

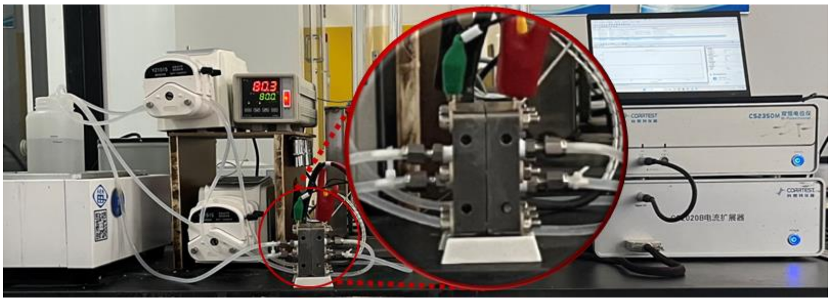

Supplementary Figure 25. Photographs of membrane electrode assembly (MEA) and related devices.

From left to right: Electric sand bath for heating electrolyte (stored in PTFE bottles), peristaltic pump for circulating electrolyte, heating device for MEA, MEA and electrochemical workstation. The effective working area of the MEA is 2×2 cm^2, and the anodes are CoFe-LDH or CoFeAl-LDH, while the cathodes are commercial Ni foam electrodes. The operating temperature is 80 ºC. And the electrolytes are 20 wt.% NaOH + 6-fold concentrated seawater.

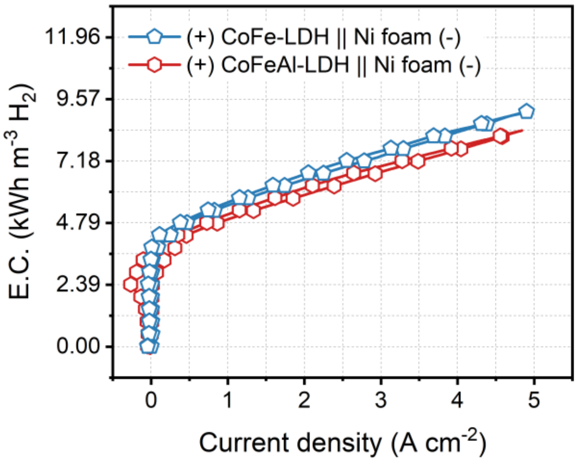

Supplementary Figure 26. Polarization curves of MEA.

The left axis shows the energy consumption (*E.C.*) of the MEA, which is calculated as:

$$E.C.(\text{kWh m}^{-3}\text{H}_2)=Voltage\ (\text{V})\times 2.39 \quad (5)$$

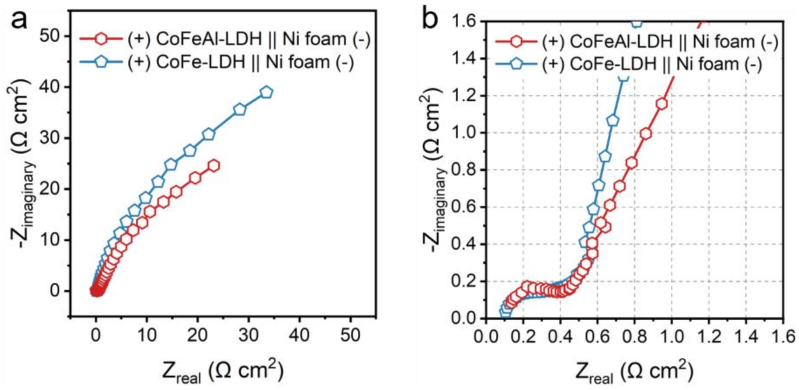

Supplementary Figure 27. (a) Electrochemical impedance spectroscopy (EIS) of MEA and (b) local amplification image.

The MEA exhibited a low ohmic resistance (0.11 Ω), which means that at high current densities, the system will not experience excessive energy losses due to ohmic polarization.

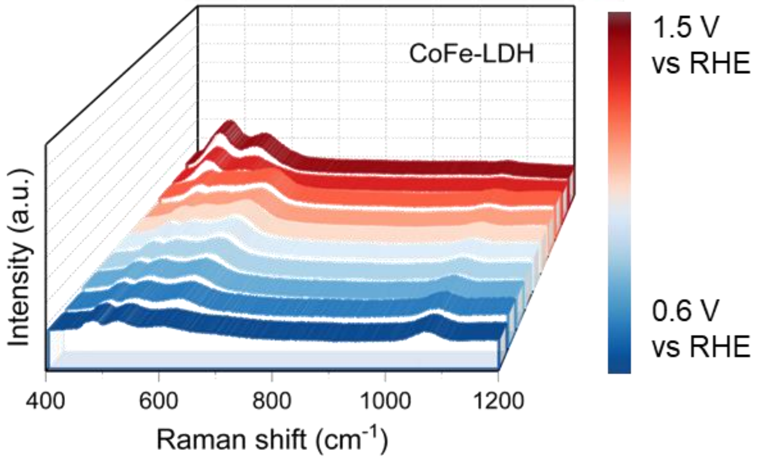

Supplementary Figure 28. Operando Raman spectrum of CoFe-LDH, potential range: open circuit potential (0.6 V vs RHE) to 1.5 V vs RHE.

Similarly, the CoFe-LDH electrode exhibited M-O vibration peaks of Co(Fe)OOH after the potential reached 1.3 V vs RHE, indicating the reconstruction and the formation of OER active sites. Moreover, the peaks of CoFeAl-LDH within the 400-700 cm^{-1} range become more pronounced compared to CoFe-LDH. As previously mentioned, this can be attributed to the etched Al^{3+} coordinating at the distal sites and adsorbing greater amounts of OH^- from the electrolyte.[20,21]

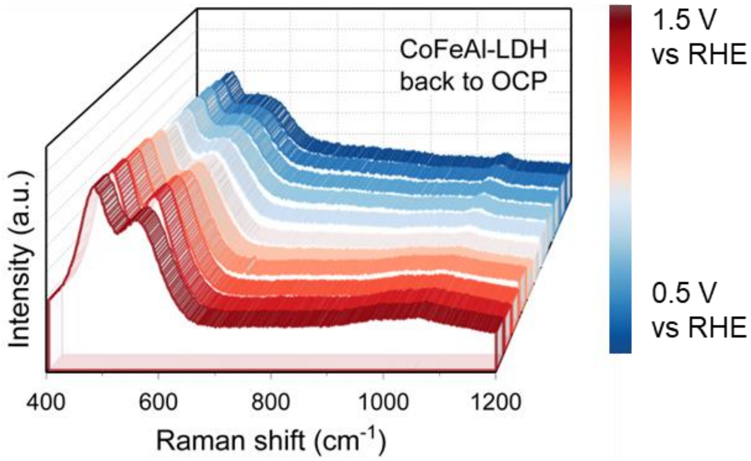

Supplementary Figure 29. Operando Raman spectrum of CoFeAl-LDH, potential range: 1.5 V vs RHE to open circuit potential (0.5 V vs RHE).

It was observed that a pair of peaks within the 400-700 cm^{-1} wavenumber range assign to $Al(OH)_n^-$ did not exhibit any significant changes. This result indicates the highly stable adsorption of $Al(OH)_n^-$ species on the electrode surface.

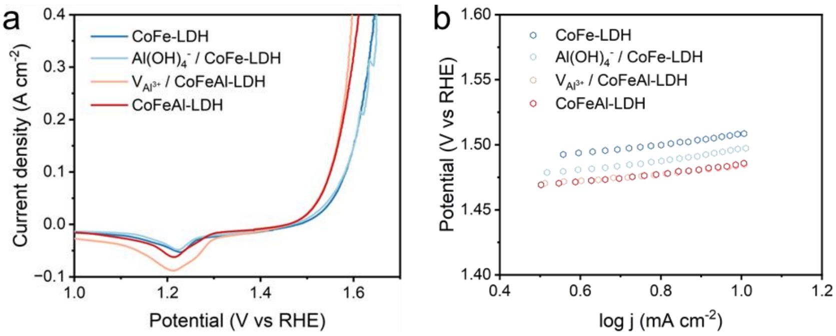

Supplementary Figure 30. (a) Polarization curves and (b) Tafel plots of CoFe-LDH before/after add $Al(OH)_n^-$ into the electrolyte (CoFe-LDH and $Al(OH)_4^-$/CoFe-LDH), and CoFeAl-LDH before/after etch Al^{3+} (CoFeAl-LDH and V_{Al3+}/CoFeAl-LDH), in an electrolyte containing 20 wt.% NaOH + saturated NaCl.

Similarly, the CoFe-LDH electrode exhibited M-O vibration peaks of Co(Fe)OOH after the potential reached 1.4V, indicating the reconstruction and the formation of OER active sites. Moreover, the peaks of CoFeAl-LDH within the 400-700 cm^{-1} range become more pronounced compared to CoFe-LDH. This can be attributed to the etched Al^{3+} coordinating at the distal sites and adsorbing greater amounts of OH^- from the electrolyte.

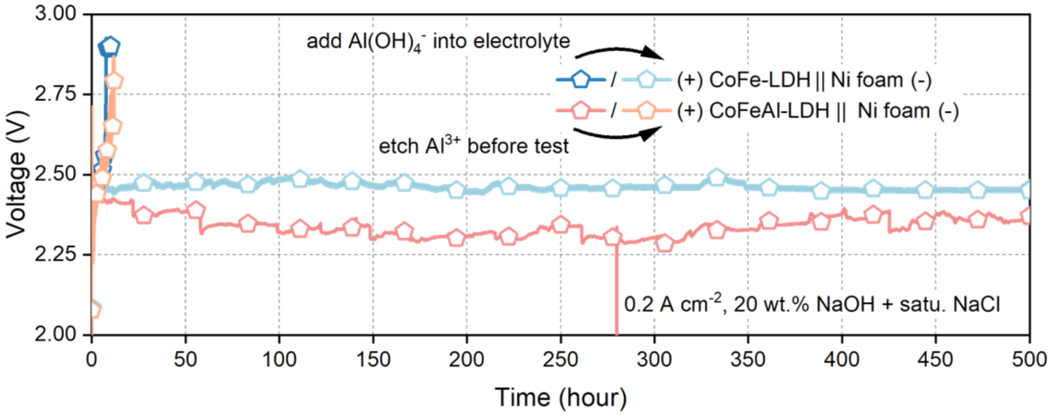

Supplementary Figure 31. CP response at 0.2 A cm^{-2} of CoFe-LDH before/after add $Al(OH)_n^-$ into the electrolyte, and CoFeAl-LDH before/after etch Al^{3+}, in an electrolyte containing 20 wt.% NaOH + satu. NaCl.

Following the introduction of $Al(OH)_n^-$ to the electrolyte, the stability of CoFe-LDH saw a notable improvement, exhibiting less than 0.29% performance degradation after a 500-hour stability test at 0.2 A cm^{-2}. In contrast, in the absence of $Al(OH)_n^-$, degradation reached 16.41% in just 10 hours. Additionally, the stability of CoFeAl-LDH experienced a significant decline after the removal of Al^{3+}.

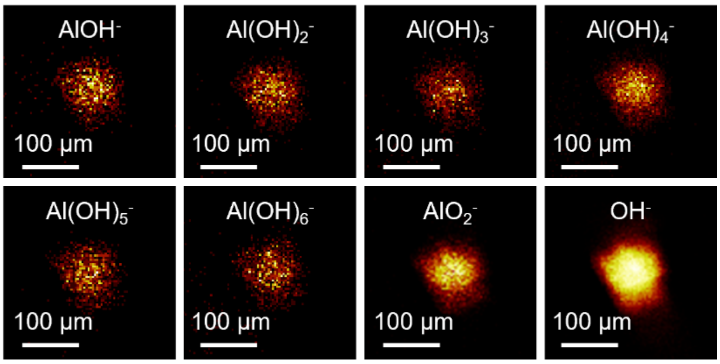

Supplementary Figure 32. TOF-SIMS mapping of $Al(OH)_n^-$ and OH^- fragments from CoFeAl-LDH electrode after activation in 20 wt.% NaOH + satu. NaCl.

It is evident that the distal Al^{3+} with different OH- coordination numbers are dispersed homogenously. The concentrations of AlO_2^- and $Al(OH)_4^-$ are relatively higher than other $Al(OH)_n^-$, which is consistent with previous reports. Note that in TOF-SIMS testing, the symbol "-" simply denotes the type of charge carried by the ion cluster and does not differentiate between the number of charges.

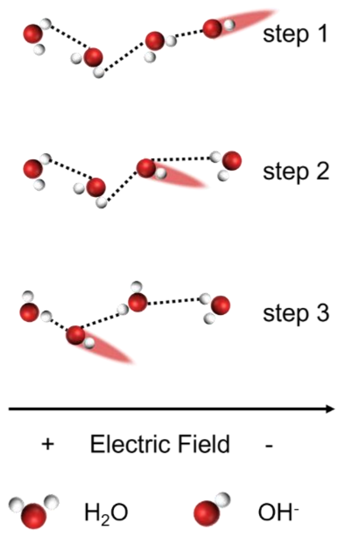

Supplementary Figure 33. The Grotthuss transfer mechanism of OH^-.

The transmission of OH^- in the aqueous electrolyte is dependent on the network of hydrogen bonds formed between neighboring water molecules, commonly known as the Grotthuss transfer mechanism. This mechanism facilitates the efficient transmission of OH^- to reach hydroxide ion surrounded $Al(OH)_n^-$.

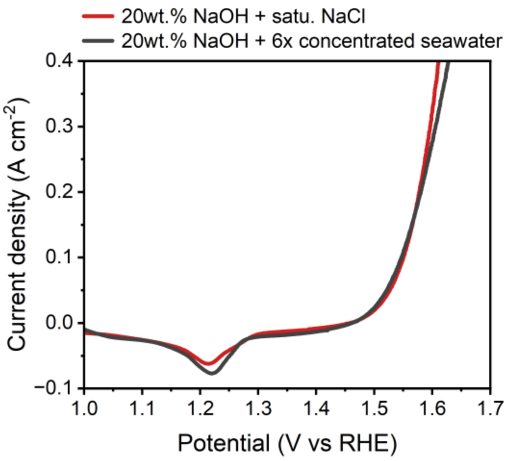

Supplementary Figure 34. The polarization curves of CoFeAl-LDH in 20 wt.% NaOH containing saturated NaCl and 6-fold concentrated seawater, respectively.

After employing the electrolyte containing 6-fold concentrated seawater, there was no significant change in the overpotential of CoFeAl-LDH at low current densities. Specifically, it required only 252 mV to achieve a current density of 10 mA cm^{-2} (compared to 256 mV in the electrolyte containing satu. NaCl). The difference in performance was primarily reflected in the rate of current growth, which may have been caused by enhanced adsorption of Cl^-/Br^- ions at higher voltages.

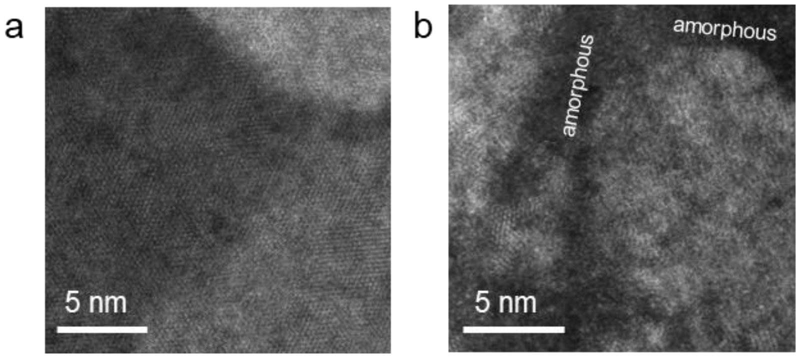

Supplementary Figure 35. HAADF-STME images of (a) CoFeAl-LDH $_{\text{before activation}}$, and (b) CoFeAl-LDH $_{\text{after activation}}$.

In contrast to the well-ordered lattice firnges observed in CoFeAl-LDH $_{\text{before activation}}$, the lattice fringes of CoFeAl-LDH$_{\text{after activation}}$ were found to be more disordered, with notable presence of amorphous regions. This phenomenon suggests a reduced crystallinity in CoFeAl-LDH $_{\text{after activation}}$.

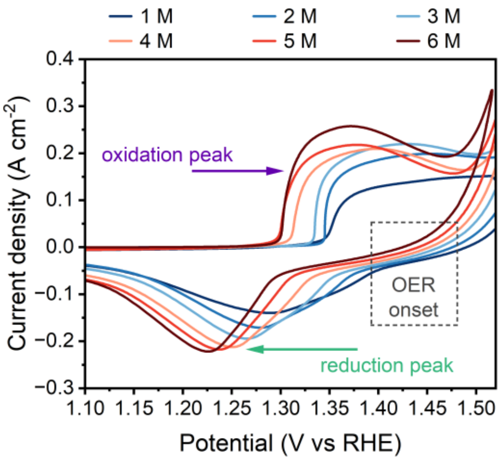

Supplementary Figure 36. Cyclic voltammetry curves of CoFeAl-LDH in the electrolytes containing different NaOH concentration.

With the gradual increase in NaOH concentration in the electrolyte from 1 M to 6 M (approximately 20 wt.%), the redox peak positions shift towards lower potentials. In addition, it is evident that the onset potential decreases with the NaOH concentration increase. This observation indicates that variations in OH^- concentration impact the reaction rate and charge transfer processes at the electrode surface.

Supplementary Information Tables

Supplementary Table 1. The atomic ratio of CoFeAl-LDH, detected by ICP-OES.

Element	Co	Fe	Al
Atomic ratio (%)	49.40	24.53	26.07

Supplementary Table 2. The stability of CoFeAl-LDH compared with some recently reported electrodes.

Material	Electrolyte	Current density (A cm^{-2})	Time (h)	Ref.
Fe-Ni_2Pv	1 M KOH + seawater	0.113	100	12
CoFe-Ni_2P	6 M KOH + seawater	1	350	13
Cr_2O_3/CoO_x	Seawater	0.5	100	14
RuNiMo	1 M KOH + 2.0 M NaCl	0.5	300	15
$Co_{3-x}Pd_xO_4$	Seawater	1	28	16
NiIr-LDH	1 M KOH + seawater	0.5	650	17
NiFe-LDH@Ag	1 M NaOH + 2.5 M NaCl	0.4	450	18
CoFePBA/Co_2P	20wt% NaOH + satu. NaCl	2	100	19
This work	20wt.% NaOH + satu. NaCl	0.2	1000	
		0.5	1000	
		1	1000	
	20wt.% NaOH + 6-fold concentrated seawater	1	500	
		1.5	350	
		2	350	

Supplementary Table 3. The ratio of Al^{3+} etched during the activation process and the total Al content in the electrode.

The concentration (Conc.) of Al^{3+} was determined through ICP-MS analysis, which involved the following steps:

(1) The mass of CoFeAl LDH grown was determined by subtracting the weight of nickel foam before and after growth, resulting in a value of 6.8 mg. Equal amounts of CoFeAl-LDH powder were dissolved in 20 mL of 0.5 M HNO_3 and subjected to ICP-MS analysis, which revealed a total Al^{3+} mass of 0.46 mg in the electrode.

(2) CoFeAl-LDH was activated, and 5 mL electrolyte samples were taken at 2, 4, 6, 8, and 10 hours. These samples were centrifuged to remove any insoluble material, then 5 mL of 7 M HNO_3 was added. The resulting solution was diluted with 10 mL of deionized water, and subjected to ICP-MS analysis.

(3) 6.8 mg of CoFeAl-LDH powder was added to 100 mL of 20 wt.% NaOH + satu. NaCl solution and stirred magnetically for 10 hours at room temperature. The solution was then centrifuged to remove any insoluble material, and 5 mL of the resulting solution was combined with 5 mL of 7 M HNO_3. The solution was then diluted with 10 mL of deionized water and subjected to ICP-MS analysis.

Samples	Conc. (ppm)	Mass (mg)	Ratio (%)
2 h	0.265	0.106	23.09%
4 h	0.361	0.144	31.41%
6 h	0.387	0.155	33.71%
8 h	0.405	0.162	35.28%
10 h	0.413	0.165	35.94%
Etch 10h	0.715	0.286	62.38%
Total	2.295	0.459	100.00%

Supplementary Table 4. TOF-SIMS relative abundance of $Al(OH)_n^-$, OH^- and Cl^- from CoFeAl-LDH electrode surface.

The relative intensity of the Al^{3+} sites enriched with OH^- is determined by summing the products of various $Al(OH)_n^-$ species and their respective n values. It is important to note that when AlO_2^- combines with two H_2O molecules, it is essentially the same as $Al(OH)_4^-$. Furthermore, in TOF-SIMS testing, the symbol "-" simply denotes the type of charge carried by the ion cluster and does not differentiate between the number of charges.

Species	Relative abundance	OH^- Relative abundance	Total
$AlOH^-$	1.182 k	1.182 k	
$Al(OH)_2^-$	1.629 k	3.258 k	
$Al(OH)_3^-$	1.481 k	4.443 k	
$Al(OH)_4^-$	2.957 k	11.828 k	88.148 k
$Al(OH)_5^-$	1.339 k	6.695 k	
$Al(OH)_6^-$	1.357 k	8.142 k	
AlO_2^-	13.150 k	52.600 k	
OH^-	71.040 k	71.040 k	71.040 k
Cl^-	29.000 k	29.000 k	29.000 k

Supplementary Table 5. The concentration of ions in 6-fold concentrated seawater and real seawater.

The composition of seawater is exceedingly complex. Notably, the presence of Ca^{2+} and Mg^{2+} ions pose a challenge due to their propensity to precipitate upon the introduction of NaOH, necessitating their prior removal. Furthermore, during the process of electrolysis, Cl^- and Br^- ions act synergistically to induce severe corrosion on the anode. This corrosive effect intensifies with the concentration of seawater, leading to more severe corrosion. In addition, seawater also contains small amounts of ions such as SO_4^{2-} and CO_3^{2-}. When these ions adsorb onto the anode, they can enhance its stability.

Composition	6x concentrated seawater	Real seawater	Impact
Cl^-	119859.36 ppm	19365.75 ppm	Negative
Na^+	61302.81 ppm	10765.89 ppm	No impact
Br^-	1045.815 ppm	171.254 ppm	Negative
SO_4^{2-}	9384.135 ppm	2712.55 ppm	Positive
K^+	2006.51 ppm	433.32 ppm	No impact
Ca^{2+}	822.765 ppm	418.97 ppm	Negative
Mg^{2+}	7019.895 ppm	1299.97 ppm	Negative

Supplementary Information Reference

1. Dionigi, F. *et al.* Design criteria, operating conditions, and nickel-iron hydroxide catalyst materials for selective seawater electrolysis. *ChemSusChem* **9**, 962–972 (2016).
2. Li, P. *et al.* Common-ion effect triggered highly sustained seawater electrolysis with additional NaCl production. *Research* **2020**, 1–9 (2020).
3. Sharma, S. K. *Green corrosion chemistry and engineering: opportunities and challenges.* (Wiley. VCH, 2011).
4. Niu, S. *et al.* Fabrication of uniform Ru-doped $NiFe_2O_4$ nanosheets as an efficient hydrogen evolution electrocatalyst. *Chem. Commun.* **55**, 14649–14652 (2019).
5. Liu, H. *et al*. Effect of Al content on the structure of Al-substituted goethite: a micro-Raman spectroscopic study. *J. Raman Spectrosc.* **44**, 1609–1614 (2013).
6. Sakita, A. M. P. *et al*. Pulse electrodeposition of CoFe thin films covered with layered double hydroxides as a fast route to prepare enhanced catalysts for oxygen evolution reaction. *Appl. Surf. Sci.* **434**, 1153–1160 (2018).
7. Ram, S. *et al.* Infrared spectral study of molecular vibrations in amorphous, nanocrystalline and $AlO(OH)\cdot\alpha H_2O$ bulk crystals. *Infrared Phys. Technol.* **42**, 547–560 (2001).
8. Bonnelle, J. P. *et al*. Influence de la polarisation des liaisons sur les spectres esca des oxydes de cobalt. *J. Electron Spectros. Relat. Phenomena* **7**, 151–162 (1975).
9. Xu, W. *et al*. Superwetting electrodes for gas-involving electrocatalysis. *Acc. Chem. Res.* **51**, 1590–1598 (2018).
10. Liu, W. *et al.* Synergistic N-doping and heterointerface engineering in W_2C/W nanoarrays

enable pH-universal hydrogen evolution catalysis. *ACS Appl. Eng. Mater.* **1**, 861–867 (2023).

11. De Backer, A. *et al*. Atom counting in HAADF STEM using a statistical model-based approach: Methodology, possibilities, and inherent limitations. *Ultramicroscopy* **134**, 23–33 (2013).
12. Liu, X. *et al.* Manipulating electron redistribution in Ni_2P for enhanced alkaline seawater electrolysis. *Adv. Mater.* 2307395 (2023).
13. Huang, C. *et al.* Functional bimetal co-modification for boosting large-current-density seawater electrolysis by inhibiting adsorption of chloride ions. *Adv. Energy Mater.* **13**, 2301475 (2023).
14. Guo, J. *et al.* Direct seawater electrolysis by adjusting the local reaction environment of a catalyst. *Nat Energy* **8**, 264–272 (2023).
15. Kang, X. *et al.* A corrosion-resistant RuMoNi catalyst for efficient and long-lasting seawater oxidation and anion exchange membrane electrolyzer. *Nat Commun* **14**, 3607 (2023).
16. Wang, N. *et al.* Strong-proton-adsorption Co-based electrocatalysts achieve active and stable neutral seawater splitting. *Adv. Mater.* 2210057 (2023).
17. You, H. *et al.* Monolayer NiIr-layered double hydroxide as a long-lived efficient oxygen evolution catalyst for seawater splitting. *J. Am. Chem. Soc.* **144**, 9254–9263 (2022).
18. Xu, W. *et al.* Ag nanoparticle-induced surface chloride immobilization strategy enables stable seawater electrolysis. *Adv. Mater.* 2306062 (2023).
19. Liu, W. *et al.* Ferricyanide armed anodes enable stable water oxidation in saturated saline water at 2 A/cm^2. *Angew. Chem.* **135**, e202309882 (2023).
20. Qiu, Z. *et al.* In operando Raman investigation of Fe doping influence on catalytic NiO intermediates for enhanced overall water splitting. *Nano Energy* **66**, 104118 (2019).
21. Liu, W. *et al.* Investigation on the decomposition process of sodium aluminate solution by

spectroscopic and theoretical calculation. *J. Mol. Liq*. **261**, 115–122 (2018).
